# Taste Perception of Sweet, Sour, Salty, Bitter, and Umami and Changes Due to l-Arginine Supplementation, as a Function of Genetic Ability to Taste 6-*n*-Propylthiouracil

**DOI:** 10.3390/nu9060541

**Published:** 2017-05-25

**Authors:** Melania Melis, Iole Tomassini Barbarossa

**Affiliations:** Department of Biomedical Sciences, Section of Physiology, University of Cagliari, Monserrato, Cagliari 09042, Italy; melaniamelis@unica.it

**Keywords:** taste perception, PROP taste status, l-arginine

## Abstract

Behavioral reaction to different taste qualities affects nutritional status and health. 6-*n*-Propylthiouracil (PROP) tasting has been reported to be a marker of variation in taste perception, food preferences, and eating behavior, but results have been inconsistent. We showed that l-Arg can enhance the bitterness intensity of PROP, whilst others have demonstrated a suppression of the bitterness of quinine. Here, we analyze the taste perception of sweet, sour, salty, bitter, and umami and the modifications caused by l-Arg supplementation, as a function of PROP-taster status. Taste perception was assessed by testing the ability to recognize, and the responsiveness to, representative solutions of the five primary taste qualities, also when supplemented with l-Arg, in subjects classified as PROP-tasting. Super-tasters, who showed high papilla density, gave higher ratings to sucrose, citric acid, caffeine, and monosodium l-glutamate than non-tasters. l-Arg supplementation mainly modified sucrose perception, enhanced the umami taste, increased NaCl saltiness and caffeine bitterness only in tasters, and decreased citric acid sourness. Our findings confirm the role of PROP phenotype in the taste perception of sweet, sour, and bitter and show its role in umami. The results suggest that l-Arg could be used as a strategic tool to specifically modify taste responses related to eating behaviors.

## 1. Introduction

The ability to distinguish noxious substances from nutrient-rich food sources is essential for survival [1]. Although olfaction and vision participate in food identification, taste provides the final checkpoint for food acceptance or rejection behaviors. It is generally assumed that the sense of taste can differentiate five primary sensory qualities (sweet, sour, salty, bitter and umami). However, a sixth sensory quality has recently been proposed regarding the ability to taste fatty acids [2]. Each taste quality is considered to represent different nutritional or physiological requirements, or indicate a potential dietary risk [3]. Sweet, salty, and umami are associated with specific classes of nutrients and are perceived as pleasant at low and moderate concentrations, but are avoided at high concentrations [4]. On the contrary, stimuli categorized as bitter and sour are associated with compounds that are potentially harmful, and are generally regarded as innate aversions. A sour taste allows acid detection (i.e., free protons or organic acids) and is therefore important to avoid ingesting acids in excess and overloading the mechanisms that maintain the pH body. Sour is also used to maintain electrolytic balance in humans. Bitter taste is thought to guard against consuming poisons, noxious substances, or toxins, many of which taste bitter to humans [3]. The various taste qualities act synergistically to arrange appetitive responses to energy- and protein-rich food sources (sweet, fatty acids, and umami), control intake of an adequate amount of sodium (low-salt taste), and warn against the ingestion of toxic substances or excess salt (bitter, sour, and high-salt tastes) [3]. In addition, gustatory information gives us the possibility to make a choice among different foods and choose the most appropriate, depending on the nutritional needs of the moment.

Taste perception occurs when water-soluble chemicals in the mouth contact the epithelial cells of the taste buds [3]. Perception of the different taste qualities is mediated by diverse mechanisms which are located in the cells belonging to three functional classes [3]. Heterodimer G-protein-coupled receptors (GPCRs) mediate the sweet and umami transduction: Taste receptor type 1 member 2 (T1R2) + T1R3 sweet [5,6,7] and T1R1 + T1R3 umami [8,9], although other candidate receptors for sweet and umami may exist [10,11,12]. The family of GPCRs T2Rs, respond to a diversity of bitter taste molecules [13,14,15,16,17]. 

Taste perception varies greatly among individuals, strongly influencing food preferences and selection, and therefore nutritional status and health [18]. Although the individual differences in taste-related behaviors concern all taste qualities, in the last decades, the genetic predisposition to perceive the bitter taste of 6-*n*-propylthiouracil (PROP) has gained considerable attention as a prototypical taste stimulus and an oral marker of food preferences and eating behavior that has an impact on body composition and health [18]. This assumption is based on data showing that individuals who perceive PROP as more bitter (super-tasters), compared with those who detect PROP only at high concentration or not at all (non-tasters), are also more responsive to various oral stimuli, including other bitter-tasting compounds [19,20,21,22,23,24,25], sweet substances [26], sour chemicals [27], irritants [28,29], and fats [30,31], and they typically show lower acceptance of fruits, vegetables [32,33,34], and strong-tasting, or high-fat foods [30,31,32,35,36], lower body mass index (BMI) [18,37,38,39], plasma antioxidant status [40], and colon cancer risk [41,42]. However, other studies have not confirmed these associations [43,44,45,46,47,48], and the role of phenotype of PROP sensitivity in umami perception or acceptability for protein-rich foods have not been fully studied [49]. 

Individual differences in taste sensitivity may arise from genetic differences in taste receptors [50,51] which have been found to contribute to variations in taste-related behaviors [52]. The allelic diversity of the gene that codifies the bitter receptor TAS2R38 can explain most of the PROP phenotypic differences [53,54]. A polymorphism in the gene of a salivary protein (gustin) that has been described in the literature as a taste bud trophic factor [55], can co-operate with *TAS2R38* SNP variants in modulating the PROP taste phenotype [56], by acting on fungiform papilla density and maintenance [57]. These findings provided the first mechanistic explanation of why PROP super-taster individuals have a higher density of fungiform papillae than non-tasters, and are more responsive to a wide range of stimuli that are not mediated via the specific bitter receptor. Other salivary components have been reported to contribute to individual differences in PROP tasting. Among them, specific proteins belonging to the basic proline-rich protein family, and some amino acids highly represented in their sequences, such as l-lysine and l-arginine (l-Arg), have been associated with PROP responsiveness, depending on their concentration in saliva [58,59,60]. Besides, oral supplementation with these molecules has been shown to enhance PROP bitterness perception, mostly in individuals who have low levels of these compounds in their saliva [59]. The proposed mechanism that describes the permissive role of l-Arg in PROP perception indicates that l-Arg could act as a “carrier” of the PROP molecule in saliva. This mechanism may occur by increasing PROP solubility in saliva, and/or by increasing PROP availability to receptor binding sites by stimulating hydrogen bond formation between this amino acid and PROP [60]. These authors also showed that the supplementation with l-Arg had a similar effect on bitterness intensity of caffeine [60], which is detected via five TAS2Rs [61]. These findings suggest that the effect of l-Arg in facilitating tastant bitterness perception is probably due to an increase in the availability of these molecules at receptor sites, rather than an effect on binding of tastant with the specific receptor. Finally, l-Arg is known to suppress the bitterness of quinine by specifically blocking the T2R4 receptor [62,63,64]. These observations underscore the versatility of l-Arg in modulating the bitter taste function, which is an essential amino acid for young mammals and a conditionally essential amino acid for adults [65].

The objective of this study was to analyze taste perception of the five primary taste qualities and the possible variations due to l-Arg administration, as a function of the PROP-tasting phenotype of subjects. To this aim, we assessed taste quality identification and responsiveness to representative solutions of the five primary taste qualities (sweet, sour, salty, bitter, and umami), in subjects characterized by PROP phenotype. In order to evaluate possible variations in taste perception due to l-Arg administration, we also determined the response to a low concentration of each stimulus supplemented with l-Arg (in a 1:1 molar ratio), which has already been shown to be effective in modifying bitter perception [59,60].

## 2. Materials and Methods

### 2.1. Subjects

Sixty-seven non-smoking healthy young subjects (28 men and 39 women, age 28.3 ± 0.95 years) were recruited through public advertisements at the University of Cagliari (Monserrato, Italy) between September 2014 and January 2016. All were Caucasian and were originally from Sardinia, Italy. No statistical methods were used to pre-determine sample size, but our sample size is similar to that generally employed in the field. All had a normal BMI ranging from 18.6 to 25.3 kg/m^2^ and showed no variation in body weight larger than 5 kg over the previous 3 months. None were dieting or taking medications that might interfere with taste function. None of the subjects had food allergies, or scored high on eating behavior scales (evaluated by using the three-factor eating questionnaire) [66]. This trial was registered at ClinicalTrials.gov (identifier number: UNICADBSITB-1). The Ethical Committee of the University Hospital of Cagliari approved the study procedures that have been performed in accordance with the Declaration of Helsinki (The ethical approval code is 451/09, minutes 5/2016). All subjects were verbally informed about the procedure and the aim of the study, and reviewed and signed an informed consent form. 

### 2.2. Study Design

All subjects were tested in three sessions. In the first two sessions, on 2 consecutive days, subjects were tested by using two different psychophysical approaches (described in Section 2.3) in order to identify their PROP-taster status. In the third session, after 1-month, their taste perception of the five primary taste qualities (sweet, salty, sour, bitter, umami), and the changes due to l-Arg administration were assessed. All of them were requested to abstain from eating, drinking (except water) and using oral care products or chewing gums for at least 2 h prior to sampling tastants. Women were tested on the 6th or 7th day of their menstrual cycle to avoid oral sensitivity changes due to the estrogen phase [67,68,69,70]. Subjects had to be in the test room 15 min before the beginning of the session in order to adapt to the environmental conditions which were kept constant throughout the experimental session (23–24 °C, 40–50% relative humidity, lighting with standard solar light 15,000 lux). During the tests, the testing room was kept reasonably free from odors and with a minimum level of noise. Subjects were seated in a comfortable chair. Solutions were prepared the day before each session and stored in the refrigerator until 1 h before testing. Solutions, in 10-mL samples, were presented at room temperature. Each stimulation was preceded and followed by oral rinsing with spring water.

Before starting taste assessments, a 2-mL sample of saliva was collected from each subject and placed into an acid-washed polypropylene test tube. Saliva samples were stored at −80 °C until molecular analyses were completed, as described below.

### 2.3. PROP-Taster Status

Subjects were classified for their PROP-taster status, in 2 successive days, by using two different psychophysical approaches, the three-solution test [71], and the impregnated paper screening test [72], which have been validated in several studies [55,56,59,73]. Both approaches are highly reliable as they strongly correlate with the degree of activation of peripheral taste function [74]. In both tests, the taste-intensity rating for PROP and sodium chloride (NaCl) was collected from each subject, by using the Labeled Magnitude Scale (LMS) [75]. This scale gives subjects the freedom to rate the perceived taste intensity for each stimulus relatively to the “strongest imaginable” oral stimulus they had ever experienced in their life. Subjects were trained in the use of the LMS before testing. In the three-solution test, the taste-intensity ratings were collected for three suprathreshold PROP (0.032, 0.32, and 3.2 mmol/L) (Sigma-Aldrich, Milan, Italy) and NaCl (0.01, 0.1, 1.0 mol/L) (Sigma-Aldrich, Milan, Italy) solutions in spring water, while the impregnated paper screening test is based on the ratings of two 2-paper disks, one impregnated with PROP solution (50 mmol/L) and the other with NaCl (1.0 mol/L). In both tests, PROP and NaCl were presented to each subject in blind and in a counterbalanced order. Subjects who gave lower intensity ratings to PROP solutions than to those of NaCl, or rated the PROP disk lower than 13 mm on the LMS, were classified as PROP non-tasters, those who gave higher ratings to PROP solutions than to NaCl solutions, or rated the PROP disk higher than 67 mm on the LMS were classified as super-tasters, and those who gave similar ratings to the two stimuli, or rated PROP disk with intermediate ratings, were classified as medium-tasters. Only subjects likewise classified by the two methods were included in the study: 31.25% (*n* = 20) were classified as non-tasters; 54.69% (*n* = 35) were classified as medium-tasters; and 14.06% (*n* = 9) were classified as super-tasters. A three-way ANOVA was used to document the presence of the three taster groups (see Table 1). 

### 2.4. Sweet, Salty, Sour, Bitter and Umami Perception Assessments and Effect of l-Arg Supplementation Indistinguishable

The taste quality identification and responsiveness to stimulations with representative solutions of the five primary taste qualities (sweet, sour, salty, bitter, and umami) were determined in each subject. Two concentrations for each stimulus were chosen based on previous data [49,76] and preliminary tests: a first concentration (low) chosen to be just above the recognition threshold and a second one (high) to be clearly supra-threshold. The following supra-threshold concentrations were used: sucrose, 20 and 146 mmol/L; NaCl, 20 and 85 mmol/L; citric acid, 1.3 and 5.2 mmol/L; caffeine, 1.5 and 6.7 mmol/L; monosodium glutamate (MSG), 10 and 80 mmol/L. In order to evaluate possible variations in taste perception due to l-Arg administration, the low concentration of each stimulus was also presented supplemented with l-Arg (1:1 molar ratio l-Arg), which has previously been shown to be effective in bitter perception [59,60]. Each subject was tested for each taste quality, in a double blinded and a counterbalanced order, with three cups (10-mL samples): one containing the low concentration of the stimulus; one containing the high concentration and one containing the low concentration supplemented with l-Arg. They were instructed to swish the entire content of a cup in their mouth for 10 s and then to spit it out. The interstimulus interval was set at 10 min. For each solution, subjects first had to identify the taste quality from a list of five descriptors, i.e., sweet, sour, salty, bitter and umami (multiple forced-choice procedure) and then placed a mark on the LMS in order to indicate the perceived intensity rating.

### 2.5. Density of Fungiform Papillae

Fungiform papillae density was measured according to Melis et al. [57]. Briefly, the anterior tongue surface was dried by gently blotting with filter paper and the area was stained by placing a filter paper circle (6 mm in diameter) impregnated with a blue food dye (E133, Modecor Italiana, Varese, Italy). Several digital images were taken from each subject using a Nikon Coolpix P520 (Centro Ufficio Service, Roma, Italy) (18.1 megapixel) and the best ones were analyzed by Adobe Photoshop CS2 version 9.0 software (Adobe Systems Incorporated, San Jose, CA, USA). The fungiform papillae in the stained area were identified by their mushroom-shape and elevated structure [77], and distinguished from filiform papillae by their lighter staining with food dye [57,78,79]. The fungiform papillae were separately identified and counted by three observers, and the final measurement for each subject was based on their consensus. The density (cm^−2^) was calculated.

### 2.6. Molecular Analyses

DNA was extracted from saliva samples using the QIAamp^®^ DNA Mini Kit (QIAGEN S.r.l., Milan, Italy) according to the manufacturer’s instructions. Purified DNA concentration was estimated by measuring the optical density at 260 nm with an Agilent Cary 60 UV-Vis Spectrophotometer (Agilent technologies Australia (M) Pty Ltd., Victoria, Australia).

Subjects were genotyped for three SNPs at base pairs 145 (C/G), 785 (C/T), and 886 (G/A) of the *TAS2R38* locus, which gives rise to three non-synonymous coding exchanges (proline to alanine at residue 49, alanine to valine at residue 262 and valine to isoleucine at residue 296), resulting in the two major haplotypes, PAV (the dominant taster variant) and AVI (the non-taster recessive one) and three rare haplotypes (AAI, AAV, and PVI). The polymerase chain reaction was employed to amplify the short region of the *TAS2R38* locus, including the first polymorphisms of interest (rs713598); a 221 bp fragment was amplified with forward 5′CCTTCGTTTTCTTGGTGAATTTTTGGGATGTAGTGAAGAGGCGG-3′ and reverse 5′-AGGTTGGCTTGGTTTGCAATCATC-3′ primers. Amplified samples were digested with *Hae*III, according to our previous work [57]. For the rs1726866 and rs10246939 SNPs, TaqMan^®^ SNP Genotyping Assay (C_9506827_10 for the rs1726866 assay and C_9506826_10 for the rs10246939 assay; Applied Biosystems by Life-Technologies Italia, Europe BV) was used [80,81,82] according to the manufacturer’s specifications. Replicates and positive and negative controls were included in all reactions. 

Molecular analysis identified nine subjects who were PAV homozygous for *TAS2R38* locus, 31 who were heterozygous, and 24 who were AVI homozygous. Three subjects with a rare haplotype of *TAS2R38* were excluded. 

### 2.7. Statistical Analyses

Fisher’s exact test was used to analyze the distribution of subjects who perceived no taste, recognized the taste or described a different quality for each taste stimulus, and to compare the differences due to supplementation with l-Arg, also as function of PROP-taster status. Repeated-measures ANOVA was used to compare the differences of taste intensity evoked by the stimulation with the low and high concentration of each taste stimulus, and to analyze the differences due to supplementation with l-Arg, according to taster status or *TAS2R38* genotypes. One-way ANOVA was used to analyze differences in response to each concentration of each stimulus according to the PROP-taster status or *TAS2R38* genotypes, and the density of fungiform papillae (*n*/cm^2^) related to PROP-taster status. Post hoc comparisons were conducted with the Fisher’s least significant difference (LSD) test. Statistical analyses were conducted using STATISTICA for WINDOWS (version 7; StatSoft Inc., Tulsa, OK, USA) with 95% confidence interval. *p* values < 0.05 were considered significant.

## 3. Results

### 3.1. Perception of Sweet, Salty, Sour, Bitter, and Umami and PROP Phenotype

Based on the identification of the low concentration of sucrose, 28.13% of subjects perceived no taste, 62.50% recognized the sweet quality, and only 9.38% described a different taste quality. All subjects correctly identified the sweet quality when they were presented with the high concentration of sucrose. Regarding the identification of the low concentrations of NaCl, citric acid, and caffeine, only a few subjects perceived no taste (NaCl, 1.56%; citric acid, 7.8%; and caffeine, 9.38%) or described a different taste quality (NaCl, 17.19%; citric acid, 15.63%; and caffeine, 3.13%), while most were able to recognize the correct taste quality (NaCl, 81.25%; citric acid, 76.56%; and caffeine, 87.5%). All subjects correctly identified the taste quality when they were presented with the high concentration of the corresponding stimulus, except for one subject who perceived no taste to the citric acid solution and one described a different taste quality when tasting the caffeine solution. In the case of stimulation with the low concentration of MSG, 11.67% of subjects perceived no taste, 43.33% recognized umami, and 45% described a different quality. When subjects were presented with the high concentration of MSG, 78.38% of them correctly identified the umami taste, the others described a different taste quality. Fisher’s exact test showed that PROP-taster status did not influence the correct identification of taste quality of each stimulus (*p* > 0.05).

**Figure 1:**
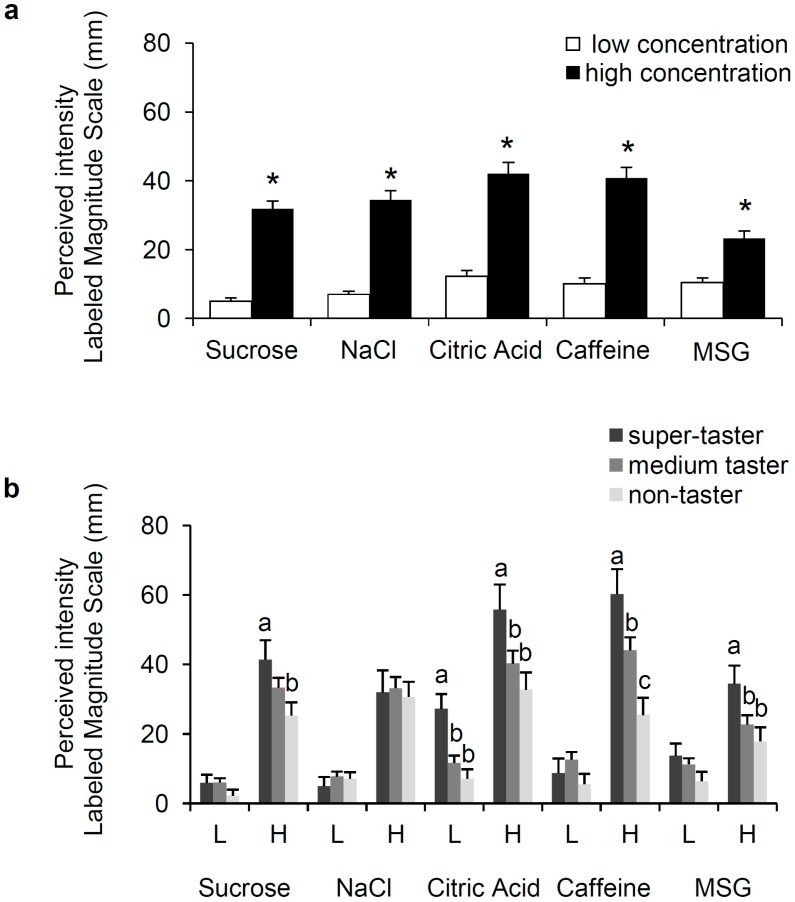
Mean values ± SEM of the taste intensity evoked by stimulation with two concentrations (low, L and high, H) of each stimulus (sucrose, 20 and 146 mmol/L; NaCl, 20 and 85 mmol/L; citric acid, 1.3 and 5.2 mmol/L; caffeine, 1.5 and 6.7 mmol/L; MSG, 10 and 80 mmol/L) (**a**). *n* = 64. The same data shown according to PROP-taster status of subjects (**b**), which was determined by the three-solution test [71] and the impregnated paper screening test [72]. Super-taster (*n* = 9, all with PAV/PAV genotype of *TAS2R38* locus), medium-taster (*n* = 35 of which 31 had PAV/AVI and four AVI/AVI genotype), non-taster (*n* = 20, all with AVI/AVI genotype). * Significant difference with respect to the corresponding value of the stimulus at low concentration (*p* < 0.00011; Fisher’s LSD test, subsequent repeated-measures ANOVA). For each solution, different letters on top of bars (a, b or c) indicate significant difference (*p* ≤ 0.05; Fisher’s LSD test; subsequent one-way ANOVA).

The taste intensity ratings evoked by stimulation with the low and high concentrations of stimuli representative of five primary taste qualities of subjects who correctly recognized the taste quality, also as a function of the PROP-tasting phenotype, are shown in Figure 1. Repeated-measures ANOVA showed that the taste intensity rating evoked by the low concentration of each stimulus was lower than that evoked by the high concentration (sucrose, *F*_(1,56)_ = 199.78, *p* < 0.00001; NaCl, *F*_(1,51)_ = 128.51, *p* < 0.00001; citric acid, *F*_(1,52)_ = 141.40, *p* < 0.00001; caffeine, *F*_(1,59)_ = 147.68, *p* < 0.00001; MSG, *F*_(1,59)_ = 65.561, *p* < 0.00001) (Figure 1a). The effect of the PROP-tasting phenotype on responsiveness to the same two concentrations of the five stimuli is shown in the Figure 1b. ANOVA showed that the sweetness intensity evoked by the high concentration of sucrose varied with PROP-taster status (*F*_(2,60)_ = 3.132, *p* = 0.05): super-tasters gave ratings significantly higher than non-tasters (*p* = 0.019; Fisher’s LSD test). No effect of PROP taster status was found on the perceived taste intensity with the low concentration of sucrose (*p* > 0.05). No difference relating to the PROP phenotype was found in response to the two concentrations of NaCl (*p* < 0.05), while ANOVA showed that the sourness intensity to both solutions of citric acid varied with taster status (low concentration, *F*_(2,50)_ = 8.115, *p* = 0.0008; high concentration, *F*_(2,60)_ = 3.491, *p* = 0.036): the values were statistically higher in super-tasters than in medium-tasters and non-tasters (lower solution, *p* ≤ 0.0017; higher solution, *p* ≤ 0.049; Fisher’s LSD test). Ratings of perceived taste intensity in response to caffeine varied with PROP-taster status only in response to the high concentration (*F*_(2,59)_ = 8.914, *p* = 0.0004): super-tasters perceived more bitterness than medium-tasters, and medium-tasters perceived a higher bitterness intensity than non-tasters (*p* ≤ 0.05; Fisher’s LSD test). Finally, the intensity of the umami taste varied with PROP-taster status in response to the high concentration of MSG (*F*_(2,57)_ = 3.173, *p* = 0.049). Post hoc comparison showed that the values determined in super-tasters were statistically higher than those of non-tasters (*p* = 0.015; Fisher’s LSD test). No changes related to taster status were found after stimulation with the low concentration of MSG (*p* > 0.05). 

The taste intensity rating evoked by the high concentration of MSG also varied as function of the genotypes *TAS2R38* (*F*_(2,30)_ = 4.255, *p* = 0.0236): the values of PAV/PAV subjects were statistically higher than those of PAV/AVI or AVI/AVI subjects (*p* = 0.014; Fisher’s LSD test) (data not shown). Individuals with PAV/AVI genotype also gave a higher rating to bitterness of the high concentration of caffeine than AVI/AVI individuals (*p* = 0.026; Fisher’s LSD test). 

### 3.2. Effect of l-Arg Supplementation on Perception of Five Taste Qualities

The solutions containing only l-Arg (1.3 mmol/L, 1.5 mmol/L and 10 mmol/L) did not evoke taste perception in all subjects. Differently, the solution containing l-Arg (20 mmol/L) was described as weakly sweet on the LMS (6.87 ± 2.38 mm) in the 34.14% of subjects, weakly bitter (5.13 ± 1.97 mm) in 4.87% while the remainder (60.99%) were not able to identify any of the tastes. The measured pH values were: 9.88 ± 0.02 for l-Arg (20 mM); 9.78 ± 0.02 for l-Arg (10 mM); 9.00 ± 0.02 for l-Arg (1.5 mM).

**Figure 2:**
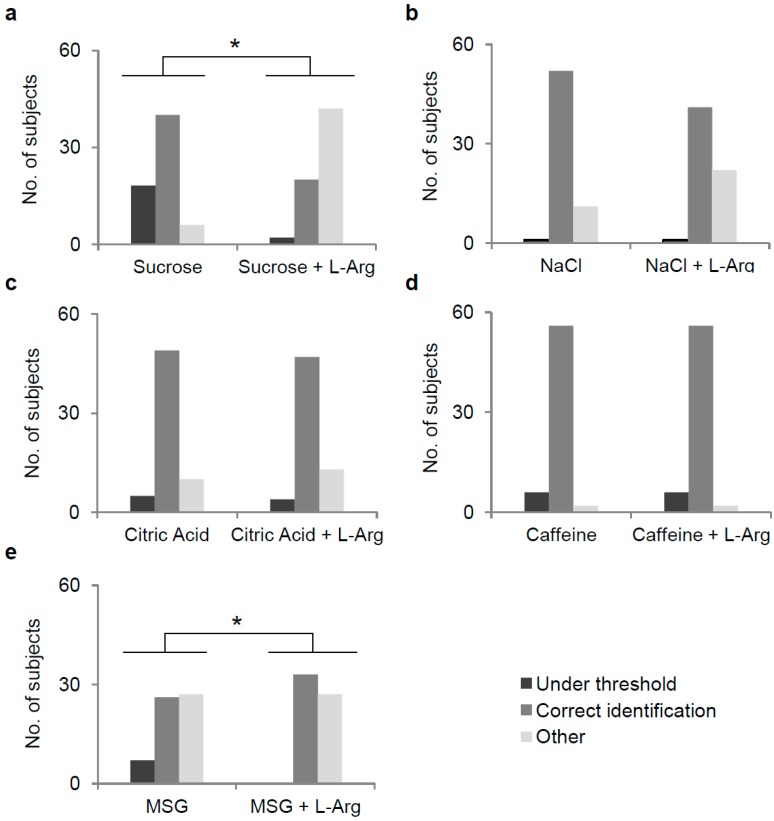
The number of subjects who perceived no taste, correctly recognized the taste, or described an incorrect taste response (Other) for the stimulus representative of each primary taste quality, presented at low concentration or supplemented with l-Arg (1:1 molar ratio l-Arg). *n* = 64. (**a**) Sucrose 20 mmol/L; (**b**) NaCl 20 mmol/L; (**c**) Citric acid 1.3 mmol/L; (**d**) Caffeine 1.5 mmol/L; and (**e**) MSG 10 mmol/L. * Significant difference (*p* < 0.00001; Fisher’s exact).

The effect of supplementation of l-Arg on identification of each taste quality is shown in Figure 2. Fisher’s exact test showed that the supplementation of l-Arg determined a modification of the sweet perception (χ^2^ = 46.46, *p* < 0.0001) (Figure 2a). Specifically, the number of subjects who correctly identified the sweet quality (31.25%) decreased, while the number of those who described a different taste quality (65.63%) increased, with respect to when they tested the solution containing only sucrose (62.50% correctly identified the sweet quality, and 9.38% described a different taste quality). Notably, the 87.8% of subjects who did not perceive sweet in the sucrose solution supplemented with l-Arg, described a bitter taste. After supplementation with l-Arg, the number of subjects who perceived no taste also decreased (3.13%), with respect to when they tested the solution containing only sucrose (28.13%).

Fisher’s exact test showed that the perception of quality did not change when NaCl, citric acid, or caffeine solutions were supplemented with l-Arg (NaCl, χ^2^ = 4.968, *p* = 0.08; citric acid, χ^2^ = 0.544, *p* = 0.76; caffeine χ^2^ = 0, *p* = 1; Fisher’s exact test) (Figure 2b–d). On the other hand, supplementation of the MSG solution with l-Arg enhanced its umami taste (χ^2^ = 7.80, *p* = 0.02; Fisher’s exact test): no subject was below threshold and the number of subjects who correctly identified the umami quality (55.00%) increased, with respect to when they tested the solution containing only MSG (43.33%) (Figure 2e). Fisher’s exact test showed that PROP-taster status did not influence the effect of l-Arg supplementation on identification of taste quality of each stimulus (*p* > 0.05).

**Figure 3:**
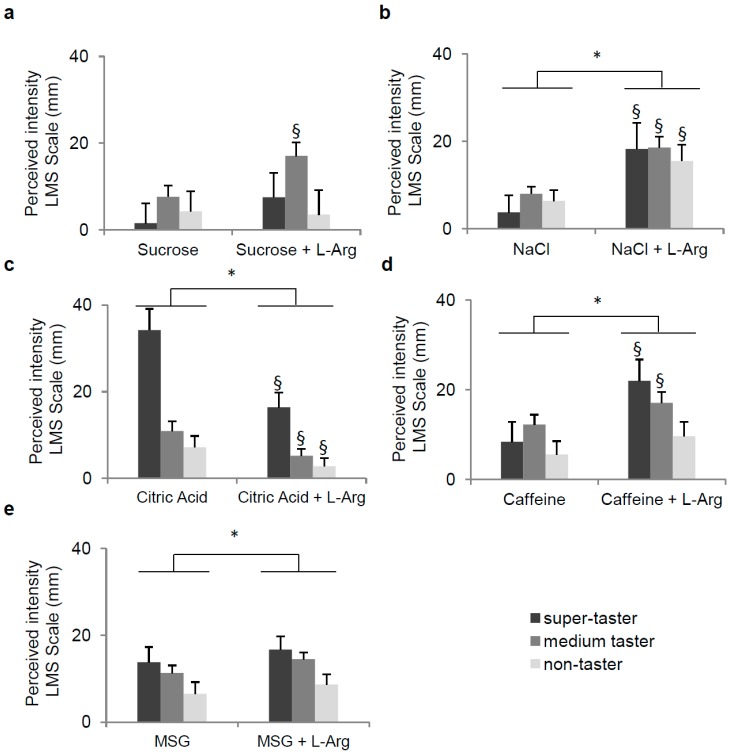
Mean values (± SEM) of taste intensity evoked by stimulation with the sucrose (20 mmol/L), NaCl (20 mmol/L), citric acid (1.3 mmol/L), caffeine (1.5 mmol/L) and MSG (10 mmol/L) and those evoked by stimulation with the same solutions supplemented with l-Arg (1:1 molar ratio) according to PROP-taster status of subjects, which was determined by the three-solution test [71] and the impregnated paper screening test [72]. (**a**) Sucrose: *n* = 21; (**b**) NaCl: *n* = 37; (**c**) Citric Acid: *n* = 46; (**d**) Caffeine: *n* = 59; and (**e**) MSG: *n* = 60. * Significant difference (*p* < 0.037; Fisher’s LSD test, subsequent repeated-measures ANOVA). ^§^ Significant difference with respect to the corresponding value of each PROP-taster group in response to each stimulus without l-Arg supplementation (*p* < 0.037; Fisher’s LSD test, subsequent repeated measures ANOVA).

Figure 3 shows the effect of l-Arg on taste intensity evoked by stimulation with sucrose, NaCl, citric acid, caffeine, and MSG in subjects who correctly detected the taste quality of each stimulus before and after l-Arg supplementation, also according to PROP-taster status. No effect of l-Arg on sweetness of sucrose was found (*p* > 0.05), even though the intensity perceived by medium-tasters after supplementation was higher than that evoked in response to stimulation with the solution with only sucrose (*p* = 0.023; Fisher LSD test, subsequent repeated-measures ANOVA) (Figure 3a). In contrast, repeated measures ANOVA showed that supplementation of l-Arg to the solutions of NaCl, caffeine, and MSG significantly increased the intensity of saltiness, bitterness, and responsiveness to umami (Figure 3b,d,e) of all subjects, while decreased that relative to sourness of citric acid (Figure 3c), with respect to those determined before supplementation (NaCl, *F*_(1,36)_ = 29.149, *p* < 0.00001; caffeine, *F*_(1,58)_ = 17.673, *p* = 0.00009; MSG, *F*_(1,57)_ = 5.571, *p* = 0.022; citric acid, *F*_(1,45)_ = 23.363, *p* = 0.00002). The analysis of the same data for each taster group showed that the supplementation of l-Arg to caffeine significantly increased bitterness intensity in super-tasters and medium-tasters (*p* < 0.0017; Fisher’s LSD test), but not in non-tasters (Figure 3d), while the effect was independent of PROP-taster status in the case of salt, acid, and umami (Figure 3b,c,e). 

No effect of l-Arg supplementation, related to *TAS2R38* genotypes, was found on perceived intensity of sucrose, NaCl, citric acid, and MSG, while the taste intensity evoked by caffeine increased after supplementation with l-Arg in PAV/AVI and AVI/AVI subjects (*p* ≤ 0.027; Fisher’s LSD test, subsequent repeated-measures ANOVA), but not statistically significant in PAV/PAV subjects (Supplementary Materials Figure S1). 

**Figure 4:**
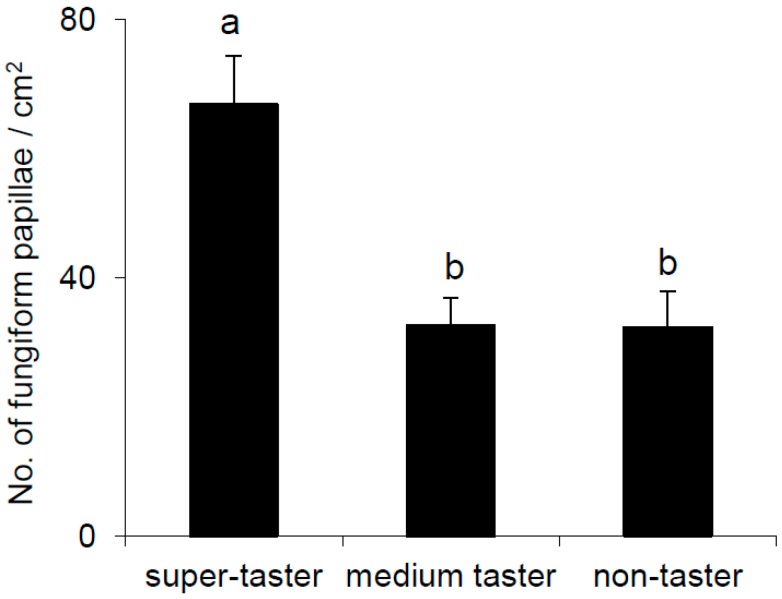
Mean values ± SEM of density of fungiform papillae (*n*/cm^2^) on the anterior part of the tongue in super-tasters (*n* = 9, all with PAV/PAV genotype of *TAS2R38* locus), medium-tasters (*n* = 35 of which 31 had PAV/AVI and 4 AVI/AVI genotype), and non-tasters (*n* = 20, all with AVI/AVI genotype). Different letters indicate significant difference (*p* ≤ 0.0006; Fisher’s LSD test; subsequent one-way ANOVA).

One-way ANOVA showed that fungiform papillae density was strongly associated with PROP-taster status (*F*_(2,48)_ = 8.541, *p* = 0.00067). Post-hoc analysis showed that the values determined in super-tasters were significantly higher than those of medium-tasters and non-tasters (*p* ≤ 0.0006; Fisher LSD test) (Figure 4). 

The fungiform papillae density was also strongly associated with *TAS2R38* locus (*F*_(2,48)_ = 5.8628, *p* = 0.0053). Post-hoc analysis showed that the values determined in subjects with genotype PAV/PAV were significantly higher than those of PAV/AVI or AVI/AVI (*p* ≤ 0.0091; Fisher LSD test) (Supplementary Materials Figure S2).

No harms or unintended effects were observed.

## 4. Discussion

The first objective of this study was to characterize the perception of the five primary taste qualities, by testing the ability to recognize and respond to two concentrations of each stimulus, and determine if these factors varied as a function of PROP-taster status. Results showed that both concentrations used for all stimuli were above threshold since they were perceived by more than 50% of subjects, who showed a higher responsiveness to the high concentration of each stimulus than to the low one. However, the low concentration of MSG likely caused taste confusion due to the unfamiliarity of subjects with this stimulus.

The role of PROP status on the perception of different taste stimuli has been extensively studied, but results have been inconsistent [18,21,22,30,31,43,83,84,85]. However, many variables that may lead to these divergent conclusions should be taken into consideration in this type of study. For example, it is well known that increasing age can diminish taste sensitivity [23,76,86,87,88], as well as papilla density [89]. Our results, which were collected in a group of young subjects selected within a limited age range, are consistent with findings showing that PROP super-tasters, compared with non-tasters, have a higher perception of other bitter-tasting compounds [19,20,21,22,23,24,25], sweeter substances [26] or sour chemicals [27] and, for the first time, we observed the influence of PROP-taster status on the fifth taste quality, umami. However, salt perception remained unchanged. Specifically, we found that PROP-taster status affected the responsiveness to high concentrations of MSG, as well as that of sucrose, citric acid, caffeine, with super-taster subjects who gave ratings higher than non-tasters and medium-tasters showed intermediate ratings which were not statistically different from those obtained in non-tasters, except in the case of caffeine. Super-tasters also perceived more taste intensity in response to the low concentration of citric acid than the other taster groups. No other differences related to PROP-taster status were found in response to the low concentration of other stimuli. PROP-taster status did not affect the responsiveness to both concentrations of NaCl, thus indicating the validity of the two psychophysical approaches used in this study, and confirming the use of this stimulus as a reference standard in psychophysical measurements when determining PROP-taster status [71]. 

Instead, no effect of PROP taster status on the ability to correctly recognize a taste quality was found. In addition, our results showed a higher density of fungiform papillae on the anterior part of the tongue of super-tasters, as compared with other groups, in accordance with previous works [26,57,78,90,91,92]. On the other hand, medium-tasters had a density of papillae no different from that of non-tasters. Our findings, which cannot be explained by haplotypic variations in *TAS2R38*, indicate that the higher capacity of super-tasters to perceive taste stimuli which are not mediated via specific bitter taste receptors is due, partly at least, to the higher density of papillae that these subjects exhibited, compared with the other taster groups. However, other authors argue against the use of papillae density in predicting taste sensitivity [93]. Based on observations underscoring the versatility of l-Arg in modulating bitter taste function [59,60,62,63,64,94], our second objective was to characterize the effect of this amino acid on the identification and responsiveness to the five taste qualities in all subjects, and to determine the role of PROP-taster status in the obtained changes. Results showed that the supplementation of l-Arg to solutions representative of the five taste qualities determined profound modifications of perception by affecting either the taste identification or the responsiveness and these modifications were different for each stimulus. Specifically, l-Arg determined a change of the perceived taste quality when it was added to sucrose solution. In fact, most subjects (61%) perceived this solution as bitter rather than sweet, although the solution containing only l-Arg was perceived as bitter by very few subjects. This result seems to suggest an effect of the l-Arg on perception of sweet similar to that of artificial sweeteners, such as stevia or sucralose, which can cause a bitter aftertaste by acting specifically on T2Rs bitter receptors [95,96]. In addition, l-Arg enhanced the sweetness of sucrose solution in those subjects who continued to perceive sweet after l-Arg supplementation. This effect seemed specific for the PROP-taster subjects. Conversely, l-Arg enhanced the taste when it was added to the MSG solution. The number of subjects who were able to perceive the umami taste increased when the supplemented solution was tested, and so increased the rating of perceived intensity, but independently from the PROP-taster status of subjects. Moreover, the supplementation of l-Arg enhanced the saltiness of NaCl independently of taster status of subjects, thereby confirming its previously demonstrated action as a salt taste enhancer [97] which could be of great importance to individuals with hypertension. Besides, it enhanced the bitterness of caffeine only in super-tasters and medium-tasters, but not in PROP non-tasters. Since bitter is the taste quality to which humans are most sensitive, the effect of l-Arg as a bitter enhancer is very interesting and should be further examined in future studies. Finally, l-Arg supplementation decreased the sourness of citric acid in all subjects [98], which is not surprising since the l-Arg is a basic amino acid. These results, which should be confirmed in a larger population (with a higher number of subjects in each PROP-taster group) and also by using other methods commonly used in taste studies, such as the general LMS [99], represent an important first step in elucidating the role of this polar amino acid in modifying the taste responses relative to the five qualities and provide a basis for further investigations, also by using different concentrations.

Although additional studies are needed also to understand the mechanism by which l-Arg determines profound modifications of taste perception, the opposite effects on perception of the sucrose solution could be the result of the positive and negative feedback that have been demonstrated in taste buds, combined to produce the sensory output transmitted to brain [3]. One may speculate that l-Arg stimulates a particular receptor cell (i.e., bitter cell), which mediates lateral inhibition via serotonin [3], suppressing the output of the adjacent sweet receptor cell. The psychophysical experiments showing that humans perceive l-Arg as bitter could support this hypothesis [100]. Although the T1R1 + T1R3 receptor is involved in L-amino acid transduction, it is not the only receptor involved in L-amino acid taste [101]. The GPCR for l-Arg has been found in mice [9], but l-Arg can also activate ionotropic glutamate receptors, causing depolarization of the taste receptor cell [102]. l-amino acids, among which l-Arg, can elicit synergistic and non-synergistic responses in a subset of taste sensory cells (i.e., salt cells) that seem to be mediated by multiple receptors [101]. Based on these findings, it is not surprising that l-Arg supplementation can also have the effect of increasing perception, which should be mediated by a different transduction mechanism. In this case, one may speculate that l-Arg amplifies the response of a particular receptor cell via a purinergic autocrine positive feedback [3].

## 5. Conclusions

The present results confirm the role of PROP-taster status on sweet, sour, and bitter responsiveness, point out its enhancing effect on umami taste, and suggest that the differences found in the three taster groups can be due, at least in part, to differences in papilla density among these individuals. In addition, our findings show that supplementation with l-Arg can selectively modify the taste responses relative to the five qualities, and suggest that this mechanism, by altering the taste properties of foods, thus making them more or less desirable, may be used as a dietetic strategic tool to optimize eating behaviors and health.

## Figures and Tables

**Table 1:** Ratings of perceived taste intensity in response to three concentrations of PROP and NaCl in the taster groups.

	Super-Tasters (*n* = 9)	Medium-Tasters (*n* = 35)	Non-Tasters (*n* = 20)
**PROP (mmol/L)**			
0.032	8.64 ± 2.00	2.86 ± 0.65	0.29 ± 0.13
0.32	46.62 ± 3.18 *	33.72 ± 2.33	2.06 ± 0.94 *
3.2	88.53 ± 4.25 *	61.22 ± 3.44	16.78 ± 2.29 *
**NaCl (mol/L)**			
0.01	1.66 ± 0.60	5.13 ± 0.95	2.34 ± 0.53
0.1	16.69 ± 2.15 *	24.12 ± 1.81	23.17 ± 2.89 *
1	57.11 ± 7.10 *	61.69 ± 3.74	60.21 ± 4.43 *

Values are means ± SEM. *n* = 64. Three-way ANOVA was used to compare PROP bitterness intensity ratings with NaCl saltiness intensity ratings across groups (*F*_(4,366)_ = 13.651; *p* < 0.00001). * Significant difference between PROP and the corresponding NaCl concentration (*p* < 0.00018; Fisher’s LSD test).

## Supplementary Materials

Supplementary material is available online at www.mdpi.com/2072-6643/9/5/541/s1.

## Data Access and Responsibility

Melania Melis and Iole Tomassini Barbarossa, had full access to all of the data in the study and take responsibility for the integrity of the data and the accuracy of the data analysis.
