# Supplementary material for: Taste Perception of Sweet, Sour, Salty, Bitter, and Umami and Changes Due to l-Arginine Supplementation, as a Function of Genetic Ability to Taste 6-n-Propylthiouracil
Source: Nutrients. 2017 May 25;9(6):541. doi: 10.3390/nu9060541 (PMC5490520; doi:10.3390/nu9060541)
Supplement: Supplementary file 1 [file nutrients-09-00541-s001.zip › nutrients-188627-supplementary.pdf]

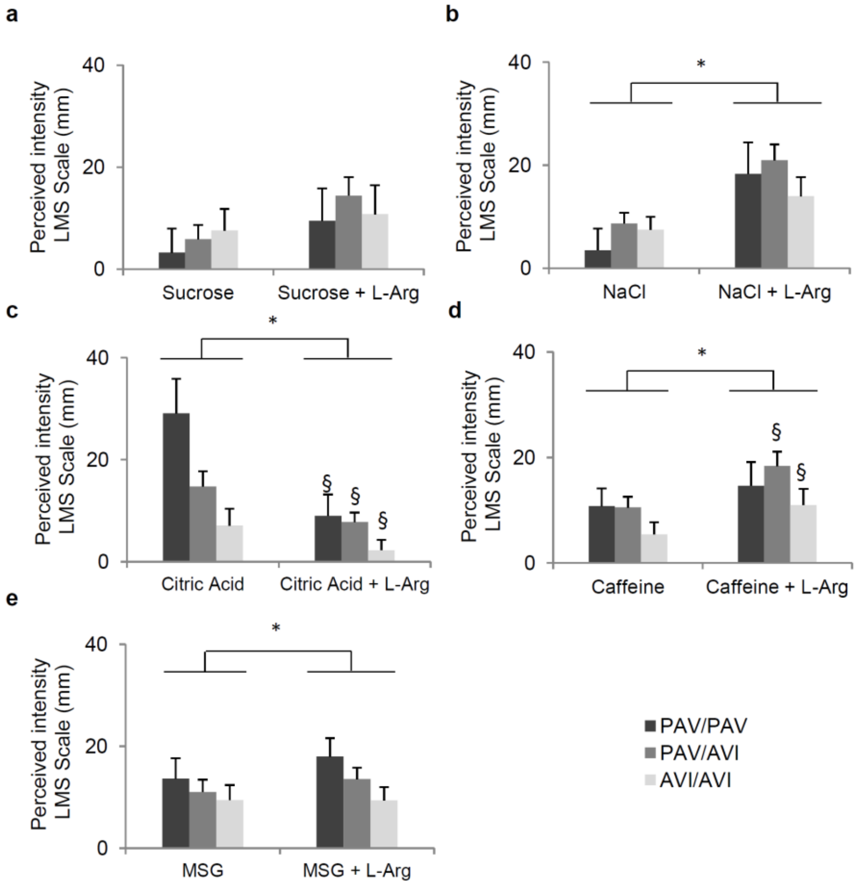

Supplemental Figure 1. Mean values (± SEM) of taste intensity evoked by stimulation with the sucrose (20 mmol/L), NaCl (20 mmol/L), citric acid (1.3 mmol/L), caffeine (1.5 mmol/L) and MSG (10 mmol/L) and those evoked by stimulation with the same solutions supplemented with L-Arg (1:1 molar ratio) according to *TAS2R38* genotypes. (a) Sucrose: $N = 21$. (**b**) NaCl: $N = 37$. (**c**) Citric Acid: $N = 46$. (**d**) Caffeine: $N = 59$. (**e**) MSG: $N = 60$. *Significant difference ($p < 0.037$; Fisher's LSD test, subsequent repeated-measures ANOVA). §Significant difference with respect to the corresponding value of each *TAS2R38* genotype in response to each stimulus without L-Arg supplementation ($p < 0.027$; Fisher's LSD test, subsequent repeated measures ANOVA).

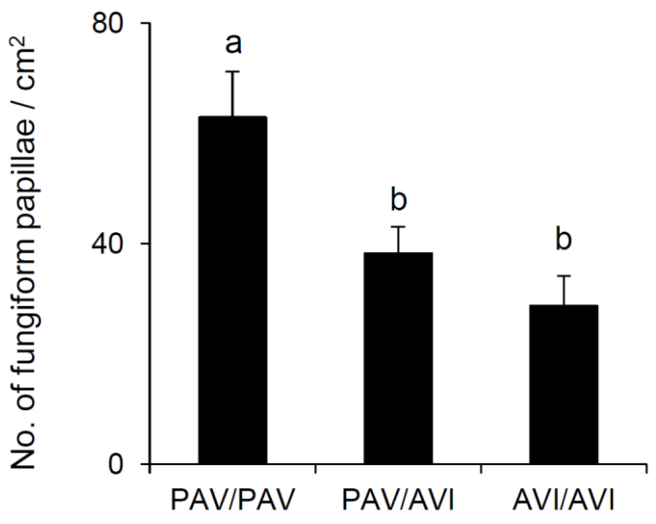

Supplemental Figure 2. Mean values ± SEM of density of fungiform papillae (n/cm^2) on the anterior part of the tongue in subjects with PAV/PAV genotype in *TAS2R38* locus (N = 9), PAV/AVI genotype (N = 31) and AVI/AVI genotype (N = 24). Different letters indicate significant difference ($p \leq 0.0091$; Fisher's LSD test; subsequent one-way ANOVA).
